# Correlation of Different Serum Biomarkers with Prediction of Early Pancreatic Graft Dysfunction Following Simultaneous Pancreas and Kidney Transplantation

**DOI:** 10.3390/jcm11092563

**Published:** 2022-05-03

**Authors:** Nora Jahn, Maria Theresa Voelker, Sven Laudi, Sebastian Stehr, Stefan Schneeberger, Gerald Brandacher, Elisabeth Sucher, Sebastian Rademacher, Daniel Seehofer, Robert Sucher, Hans Michael Hau

**Affiliations:** 1 Department of Anesthesiology and Intensive Care Medicine, University Hospital of Leipzig, 04103 Leipzig, Germany; nora.jahn@medizin.uni-leipzig.de (N.J.); theresa.voelker@medizin.uni-leipzig.de (M.T.V.); sven.laudi@medizin.uni-leipzig.de (S.L.); sebastian.stehr@medizin.uni-leipzig.de (S.S.); 2 Department of Visceral, Transplant and Thoracic Surgery, Innsbruck Medical University, 6020 Innsbruck, Austria; stefan.schneeberger@i-med.ac.at; 3 Department of Plastic and Reconstructive Surgery, Vascularized Composite Allotransplantation (VCA) Laboratory, Johns Hopkins University, Baltimore, MD 21231, USA; gbranda2@jhmi.edu; 4 Department of Oncology, Gastroenterology, Hepatology, Pneumology and Infectiology, University Hospital of Leipzig, 04103 Leipzig, Germany; elisabeth.sucher@medizin.uni-leipzig.de; 5 Department of Visceral, Transplantation, Vascular and Thoracic Surgery, University Hospital of Leipzig, 04103 Leipzig, Germany; sebastian.rademacher@medizin.uni-leipzig.de (S.R.); daniel.seehofer@medizin.uni-leipzig.de (D.S.); robert.sucher@medizin.uni-leipzig.de (R.S.); 6 Department of Visceral, Thoracic and Vascular Surgery, University Hospital and Faculty of Medicine Carl Gustav Carus, Technische Universität Dresden, 01307 Dresden, Germany

**Keywords:** simultaneous pancreas–kidney transplantation, ischemia reperfusion injury, early pancreatic graft dysfunction, early renal graft dysfunction, biomarker, lipase, C-reactive protein, procalcitonin, graft outcome, graft function

## Abstract

Background: Despite recent advances and refinements in perioperative management of simultaneous pancreas–kidney transplantation (SPKT) early pancreatic graft dysfunction (ePGD) remains a critical problem with serious impairment of early and long-term graft function and outcome. Hence, we evaluated a panel of classical blood serum markers for their value in predicting early graft dysfunction in patients undergoing SPKT. Methods: From a prospectively collected database medical data of 105 patients undergoing SPKT between 1998 and 2018 at our center were retrospectively analyzed. The primary study outcome was the detection of occurrence of early pancreatic graft dysfunction (ePGD), the secondary study outcome was early renal graft dysfunction (eRGD) as well as all other outcome parameters associated with the graft function. In this context, ePGD was defined as pancreas graft-related complications including graft pancreatitis, pancreatic abscess/peritonitis, delayed graft function, graft thrombosis, bleeding, rejection and the consecutive need for re-laparotomy due to graft-related complications within 3 months. With regard to analyzing ePGD, serum levels of white blood cell count (WBC), C-reactive protein (CRP), procalcitonin (PCT), pancreatic lipase as well as neutrophil–lymphocyte ratio (NLR) and platelet–lymphocyte ratio (PLR) were measured preoperatively and at postoperative days (POD) 1, 2, 3 and 5. Further, peak serum levels of CRP and lipase during the first 72 h were evaluated. Receiver operating characteristics (ROC) curves were performed to assess their predictive value for ePGD and eRGD. Cut-off levels were calculated with the Youden index. Significant diagnostic biochemical cut-offs as well as other prognostic clinical factors were tested in a multivariate logistic regression model. Results: Of the 105 patients included, 43 patients (41%) and 28 patients (27%) developed ePGD and eRGD following SPKT, respectively. The mean WBC, PCT, NLR, PLR, CRP and lipase levels were significantly higher on most PODs in the ePGD group compared to the non-ePGD group. ROC analysis indicated that peak lipase (AUC: 0.82) and peak CRP levels (AUC: 0.89) were highly predictive for ePGD after SPKT. The combination of both achieved the highest AUC (0.92; *p* < 0.01) in predicting ePGD. Concerning eRGD, predictive accuracy of all analyzed serological markers was moderate (all AUC < 0.8). Additionally, multivariable analysis identified previous dialysis/no preemptive transplantation (OR 2.4 (95% CI: 1.41–4.01), *p* = 0.021), donor age (OR 1.07 (95% CI: 1.03–1.14), *p* < 0.010), donor body mass index (OR 1.32 (95% CI: 1.01–1.072), *p* = 0.04), donors cerebrovascular cause of death (OR 7.8 (95% CI: 2.21–26.9), *p* < 0.010), donor length of ICU stay (OR 1.27 (95% CI: 1.08–1.49), *p* < 0.010), as well as CIT pancreas (OR 1.07 (95% CI: 1.03–1.14), *p* < 0.010) as clinical relevant prognostic predictors for ePGD. Further, a peak of lipase (OR 1.04 (95% CI: 1.02–1.07), *p* < 0.010), peak of CRP levels (OR 1.12 (95% CI: 1.02–1.23), *p* < 0.010), pancreatic serum lipase concentration on POD 2 > 150 IU/L (OR 2.9 (95% CI: 1.2–7.13), *p* = 0.021) and CRP levels of ≥ 180 ng/mL on POD 2 (OR 3.6 (95% CI: 1.54–8.34), *p* < 0.01) and CRP levels > 150 ng/mL on POD 3 (OR 4.5 (95% CI: 1.7–11.4), *p* < 0.01) were revealed as independent biochemical predictive variables for ePGD after transplantation. Conclusions: In the current study, the combination of peak lipase and CRP levels were highly effective in predicting early pancreatic graft dysfunction development following SPKT. In contrast, for early renal graft dysfunction the predictive value of this parameter was less sensitive. Intensified monitoring of these parameters may be helpful for identifying patients at a higher risk of pancreatic ischemia reperfusion injury and various IRI- associated postoperative complications leading to ePGD and thus deteriorated outcome.

## 1. Introduction

Ischemia reperfusion injury (IRI) is a major cause for early graft damage, which ultimately results in severe patient morbidity and mortality in solid organ transplantation [1,2].

The pancreatic allograft is particularly susceptible for injuries in the early reperfusion period, which among other serious complications lead to damaging effects on the grafts’ microvasculature as well as dysfunction in both, the endocrine and exocrine pancreas [3]. Notably, IRI is one of the main reasons of islet cell injury, which may be aggravated by periprocedural obstacles such as prolonged cold storage or insufficient flushing of the graft before transplantation [1,3,4,5]. Following the cold ischemic phase accompanied with reduced metabolic activity, the reperfusion and renewed onset of aerobic metabolism after reperfusion of the graft is the main mechanism for IRI [6]. Further evaluation of early indicators of IRI and a thorough investigation of cellular pathways contributing to local inflammation and reperfusion injury may help to develop preventive as well as rescue treatment strategies of IRI in solid organ transplant recipients and thereby improving graft function and outcome [7].

Of all solid organ transplantations, the pancreas graft is still most prone to development of early pancreatic graft dysfunction (ePGD), mainly induced by IRI, despite recent advances in surgical and procedural technologies, including machine perfusion instead of cold graft storage as well as refinements in immunosuppressive medications [8,9,10,11,12,13]. According to the current literature, graft-related infective complications as well as post-transplantation pancreatitis, incidents of bleeding and graft thrombosis are the main reasons for non-immunological early graft loss [12,13]. In the especially vulnerable group of SPKT patients, the identification of clinical markers, indicating an increased risk of early graft dysfunction or other IRI- associated post-transplant complications might be a valuable tool in perioperative management and therefore facilitate outcome improvement. So far, a wide range of laboratory biomarkers for the prediction of severe pancreas-related complications in the non-transplant situation were examined with good predictive values of IL-6 and CRP [14,15,16]. With regard to the transplant setting, few previous studies showed encouraging results using peak levels of serum lipase and C-reactive protein (CRP) during the first days after pancreas transplantation as potential IRI markers for graft damage [3,17]. However, it is still unclear which predictor or biomarker is superior for the prediction of IRI and consecutive ePGD and long-term outcome following pancreas transplantation.

Therefore, the goal of our current study was to evaluate and define predictive laboratory markers in clinical practice for the development of early (pancreatic and renal related) graft dysfunction in patients undergoing SPKT. Secondarily, these biomarkers were tested in combination with other known clinical factors as predictors of severity for ePGD and its application as useful biomarkers for clinical practice.

## 2. Material and Methods

### 2.1. Study Design and Study Population

The study protocol was approved by the local ethics committee of the University of Leipzig [AZ: Nr: 111–16-14032016]. From a prospectively collected electronic database, we retrospectively analyzed medical data on all patients undergoing SPKT at the University Hospital of Leipzig between 1998 and 2018. A special focus was set on the evaluation of different biochemical serum markers for their predictive value for early pancreatic graft dysfunction and renal graft dysfunction as well as graft function and outcome following SPKT.

Patients younger than 18 years, those receiving kidney transplantation alone (KTA), those receiving pancreatic re-transplantation and patients with insufficient/missing data about perioperative, intraoperative and postoperative clinical and laboratory status and outcome were excluded.

### 2.2. Outcome Analysis

Standard demographic and clinicopathological characteristics were collected and analyzed before, at the time of and after transplantation as well as in the follow-up period for each patient. Pre-transplantation data included recipient and donor characteristics such as age, sex, body mass index (BMI), donor causes of death and donor’s comorbidities and clinical course (catecholamine use, arterial hypertension, intensive care unit lengths of stay (ICU-LOS), cardiopulmonary resuscitation). Furthermore, recipient data comprised the history and duration of diabetes mellitus, time on waiting list, pre-transplantation dialysis, endocrine metabolism and information on special comorbidities (presence of coronary heart disease, peripheral arterial disease (PAD), blood pressure parameters and arterial hypertension as well as the number of antihypertensive agents). Peri- and postoperative data included information on peri- and postoperative clinical course (operation time, blood loss, cold and warm ischemia time of the pancreas as well as kidney graft, hospital stay, immunological and immunosuppressive characteristics (human leukocyte antigen (HLA) mismatches, cytomegalovirus (CMV) state, induction therapy) as well as patient and graft function and outcome. As a primary outcome parameter, the occurrence of early pancreatic graft dysfunction (ePGD) was evaluated which was defined according to previous definitions from literature [12,18,19] as typical graft-related complications within 3 months following pancreas transplantation including graft pancreatitis, pancreatic abscess/peritonitis, pancreas graft thrombosis, bleeding, pancreatic delayed graft function, acute rejection episodes and the consecutive need for re-laparotomy due to graft-related complications. Secondary outcome parameters were early renal graft dysfunction (eRGD) as well as all other outcome parameters associated with the graft function. In this context, early renal graft dysfunction (eRGD) was defined as early renal graft injury, which was manifest in the kidney as acute tubular necrosis and delayed graft function with the consequent need of for dialysis after transplantation and/or graft rejection. Pancreatic delayed graft function was defined as the need for insulin at the time of hospital discharge, but with the establishment of an insulin-independent state soon thereafter. Acute rejection episodes were suspected, if there was an abrupt increase in serum amylase/lipase and/or serum glucose levels, together with a significant drop in serum C-peptide level and/or increased serum creatinine levels and missing diuresis as well as abdominal pain associated with sonographic swelling of the graft. If possible, the diagnosis was confirmed by endoscopic biopsies of the duodenal segment of the graft. Biopsies of the kidney graft were performed, to confirm rejection. Pancreatic biopsies were not performed. Treatment of acute cellular rejection consisted of pulsed steroids or administration of anti-thymocyte globulin (ATG, 8 mg per kg bodyweight) in parallel with increased baseline immunosuppression. DGF of the kidney was defined as the requirement of dialysis in the first week following transplantation [20]. Pancreas graft failure was defined as resumed insulin therapy, removed pancreas, re-transplantation or patient death. Kidney graft failure was defined as the need for dialysis, removed kidney, re-transplantation or patient death.

### 2.3. Measurement of Biomarkers

For the evaluation of a possible predictive value for ePGD, different serum biomarkers were measured and compared between patients with ePGD and those with no ePGD.

Herein, serum samples were analyzed preoperatively and at postoperative days (POD) 1, 2, 3 and 5 after transplantation. The serum panel included standard inflammation and pancreas markers such as white blood cell count (WBC in gigaparticle per liter, Gpt/L), C-reactive protein (CRP, mg/L), procalcitonin (PCT, μg/L), serum lipase (IU/L) as well as a differential blood count to calculate the neutrophil–lymphocyte ratio (NLR) and platelet–lymphocyte ratio (PLR). Peak levels of C-reactive protein and serum lipase were also analyzed and defined as the highest serum levels within the first three days after transplantation.

### 2.4. Surgical Techniques and Immunosuppression

As described previously, pancreas and kidney grafts were procured and transplanted following the international standards and guidelines [21,22,23,24,25,26,27]. In short, the pancreas was explanted in a no-touch technique en-bloc with the spleen and duodenum. Back table preparation included removal of the spleen and peripancreatic fat. Reconstruction of the superior mesenteric and the lineal artery was performed using the donor iliac Y-graft. The pancreas was transplanted into the right iliac fossa using a standard technique with an intraperitoneal location in the right iliac fossa. The Y-graft was anastomosed to the recipient’s common iliac artery using 6-0 Prolene running sutures. The portal vein was connected to the inferior vena cava of the recipient. Exocrine drainage was carried out with a hand-sutured side-to-side duodenojejunostomy 40 cm beyond the flexure of Treitz [22,26]. All kidneys were transplanted into the contralateral iliacal fossa. Vascular anastomoses were performed to the external iliac artery and vein. The ureter was implanted into the bladder according to the Lich-Gregoir technique using a double J catheter as an intraureteral splint. Splint removal was performed 3–4 weeks after successful transplantation [27,28].

The immunosuppressive protocol consisted of an induction therapy followed by triple maintenance therapy as described previously. Shortly, for induction therapy, antithymocyte globulin (ATG, Thymoglobulin) or the interleukin-2 receptor antagonist basiliximab was used. Maintenance therapy included calcineurin inhibitors (CNI) (Cyclosporin or Tacrolimus), and/or antimetabolites (Sirolimus or Mycophenolate Mofetil (MMF)), and tapered steroids [27,28].

### 2.5. Statistical Analysis

With regard to baseline data, continuous variables are reported as mean values with standard deviation, whereas categorical variables are presented as whole numbers and percentages (%). For the analysis of baseline data, we used the appropriate statistical significance tests, including Student’s *t*-test, χ^2^, analysis of variance (ANOVA), Kruskal–Wallis and Wilcoxon–Mann–Whitney test.

To evaluate the predictive value and prognostic accuracy of the different biomarkers and clinical variables in the prediction of ePGD and eRGD, receiver operating characteristic (ROC) curves were generated, and the area under the curve (AUC) was calculated. To this end, AUCs ≥ 0.7 were defined as acceptable, AUCs ≥ 0.8 were defined as excellent and AUCs ≥ 0.9 were defined as exceptional biomarkers [29]. The optimal cut-off was identified using the Youden index (sensitivity + specifity-1), and sensitivity and specificity were subsequently calculated.

The investigation of potential clinical prognostic factors for ePGD was further carried out using logistic regression analysis. In the final multivariable model, potential risk factors with significant values in univariate analysis and/or known risk factors from the literature were selected using the backward stepwise selection procedure with adjustment for potential confounders. Results of the regression analyses were presented as odds ratio (OR) with 95% confidence interval (CI) and its corresponding *p*-value. A *p*-value < 0.05 was considered statistically significant. To determine the goodness-of-fit of the regression model, the Hosmer–Lemeshow-test (HLT) was used. The model was considered fit when *p* > 0.05 in HLT.

All statistical analyses were performed using the SPSS software (SPSS Inc., Chicago, IL, USA, version 28.0).

## 3. Results

### 3.1. Baseline Characteristics and Intraoperative Outcome

During the study period, a total of 105 SPKTs were performed and included in the current study. In total, 58 of the recipients were male and 47 were female with a mean age of 42.9 +/− 9.1 years.

Among our cohort, 43 patients (41%) and 28 patients (27%) developed ePGD and eRGD following SPKT, respectively. The mean follow-up period of the study was 151 ± 34.4 months.

Donor and recipients’ demographic and clinico-pathologic baseline characteristics with regard to patients with or without ePGD are given in Table 1. In particular, ePGD was more common in donors with cerebrovascular diseases (*p* = 0.018) and after cardiopulmonary resuscitation (*p* = 0.06). Donor age (*p* = 0.02), donor BMI (*p* < 0.01) as well as donor ICU-LOS (*p* = 0.03) were increased in patients with ePGD compared to those without. Patients with ePGD were older with a mean age of 46.1 +/− 8.6 years versus 40.9 +/− 8.7 years without ePGD (*p* < 0.01) and showed a higher presence of peripheral arterial disease (*p* = 0.08).

Table 2 shows the intra- and postoperative outcome parameters of the study groups with regard to patients with and without ePGD. In patients with ePGD, the mean cold ischemic time (CIT) of the pancreas (ePGD+: 11.9 +/− 2.4 h versus ePGD-: 10.5 +/− 2.6 h; *p* = 0.01) as well as mean length of the hospital stay (ePGD+: 62.3 +/− 34.9 days versus ePGD-: 29.5 +/− 24.8 days; *p* = 0.03) were increased compared to those without ePGD. Furthermore, in patients with ePGD the occurrence of renal delayed graft function (eRGD+: 11 (26%) versus eRGD-: 7 (11%); *p* = 0.049) and early renal graft dysfunction (eRGD+: 16 (37%) versus eRGD-: 12 (19%); *p* = 0.039) were more common. No significant differences were observed with regard to kidney CIT (*p* = 0.194), mean warm ischemic time (WIT) of the pancreas (*p* = 0.225) or the kidney (*p* = 0.454), respectively, the operating time (*p* = 0.808) as well as the incidence of CMV infections (*p* = 0.427).

### 3.2. Comparison of Biomarker Course

**Figure 1:**
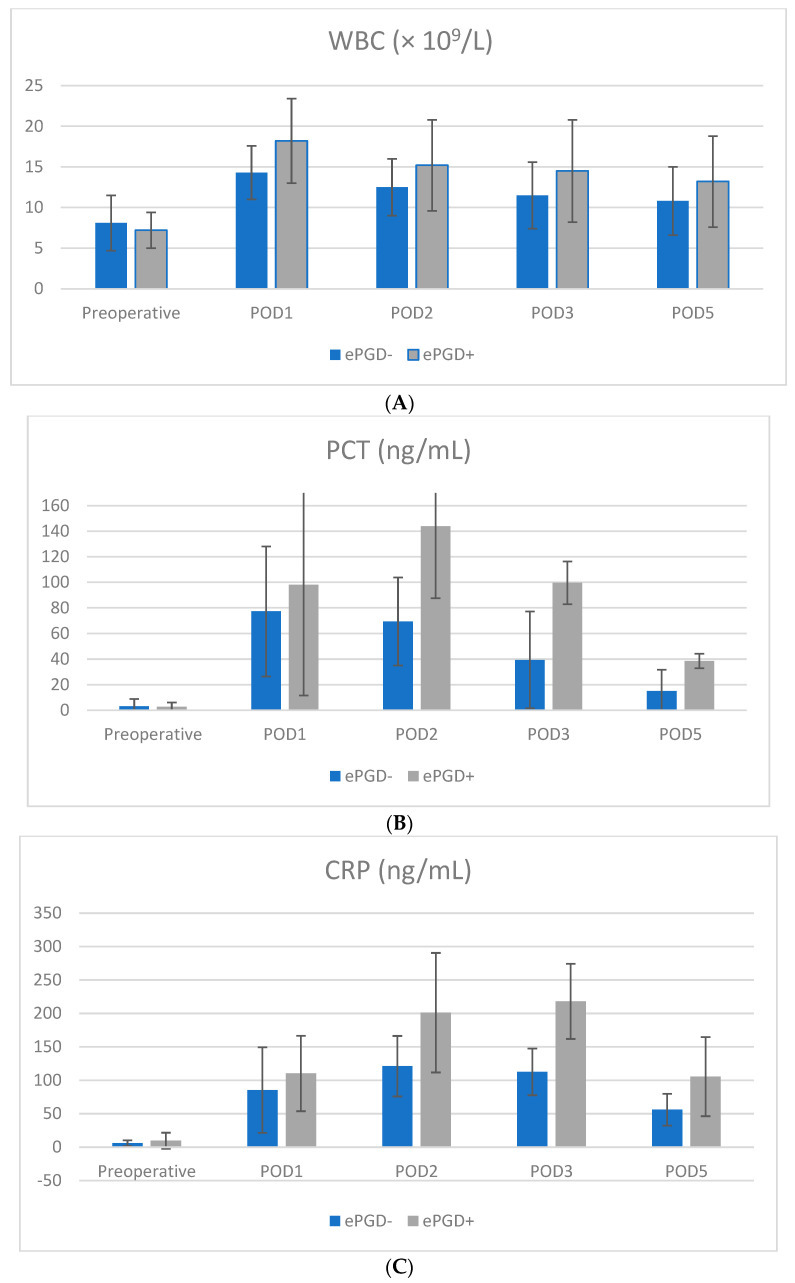

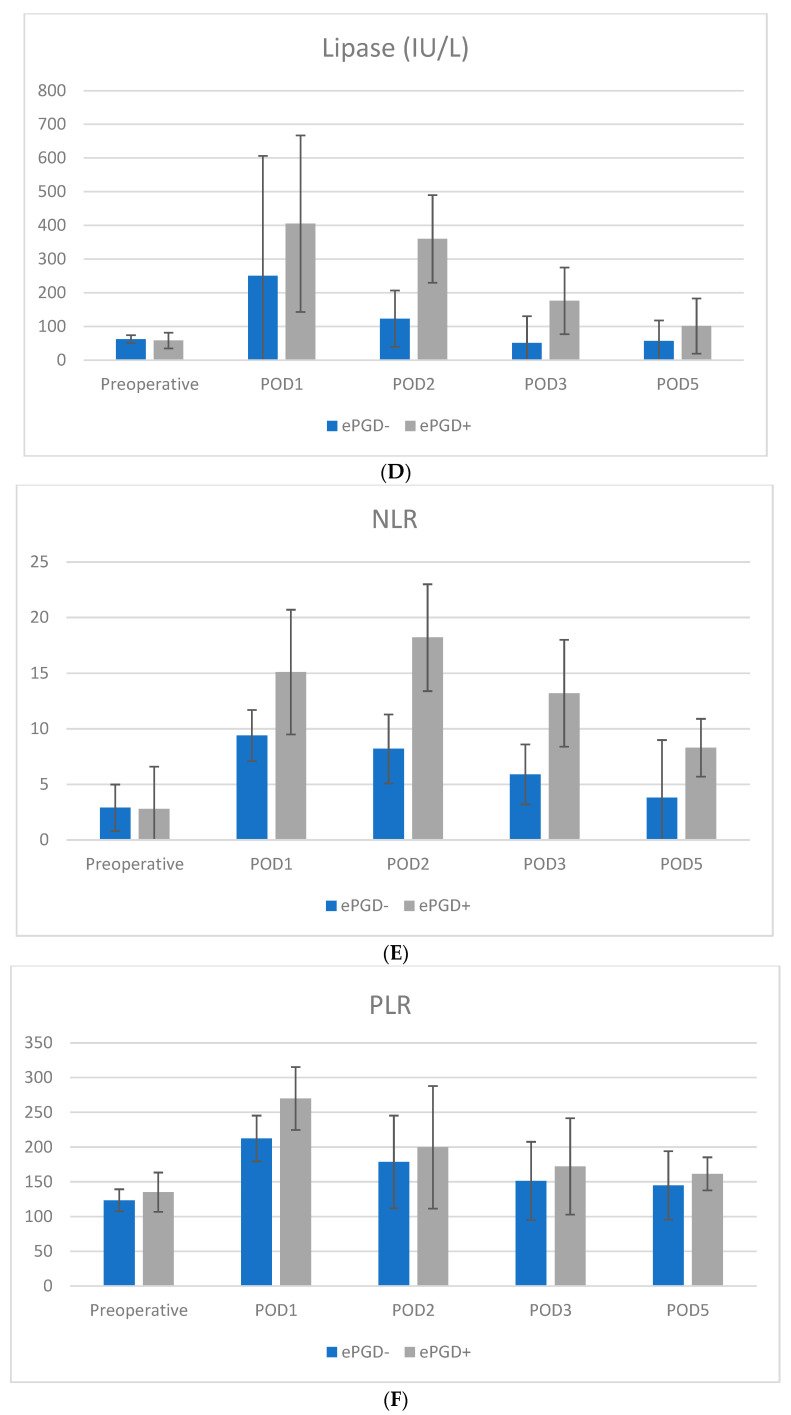
Box plots showing the distribution of WBC (**A**), PCT (**B**), CRP (**C**), Lipase (**D**), NLR (**E**) and PLR (**F**) levels in the ePGD and non-ePGD group preoperative and on POD 1, 2, 3 and 5 following SPKT.

Pre- and postoperative trends of inflammatory markers and pancreatic enzyme serum levels in patients with and without ePGD are given in Table 3 and Figure 1. Postoperative trends of WBC counts, PLR and lipase levels were similar in patients with and without ePGD with a peak on POD 1 (Figure 1A–F). CRP levels peaked on POD 3 in patients with ePGD compared to POD 2 in patients without ePGD, whereas PCT and NLR levels peaked on POD 2 in patients with ePGD compared to POD 1 in patients without ePGD. WBC counts on POD 1 and 2, CRP levels on POD 1, 2, 3 and 5, serum lipase levels on POD 1, 2 and 3, PLR levels on POD 1, NLR levels on POD 1, 2 and 3 as well as PCT levels on POD 2 were significantly higher in patients with ePGD compared to patients without ePGD (all *p* < 0.05; Table 3).

### 3.3. ROC Analysis for the Prediction of Early Graft Dysfunction

The AUC, cut-off values such as specificity and sensitivity of each analyzed biochemical marker including CRP, serum lipase, PCT, WBC, PLR and NLR levels for ePGD as well as for eRGD were determined using ROC analysis and listed in Table 4 and Table 5.

**Figure 2:**
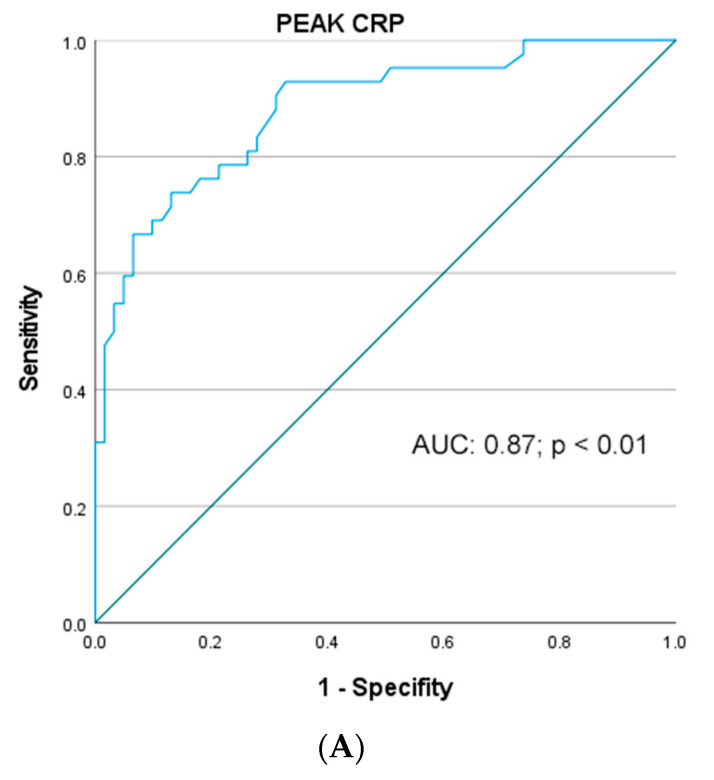

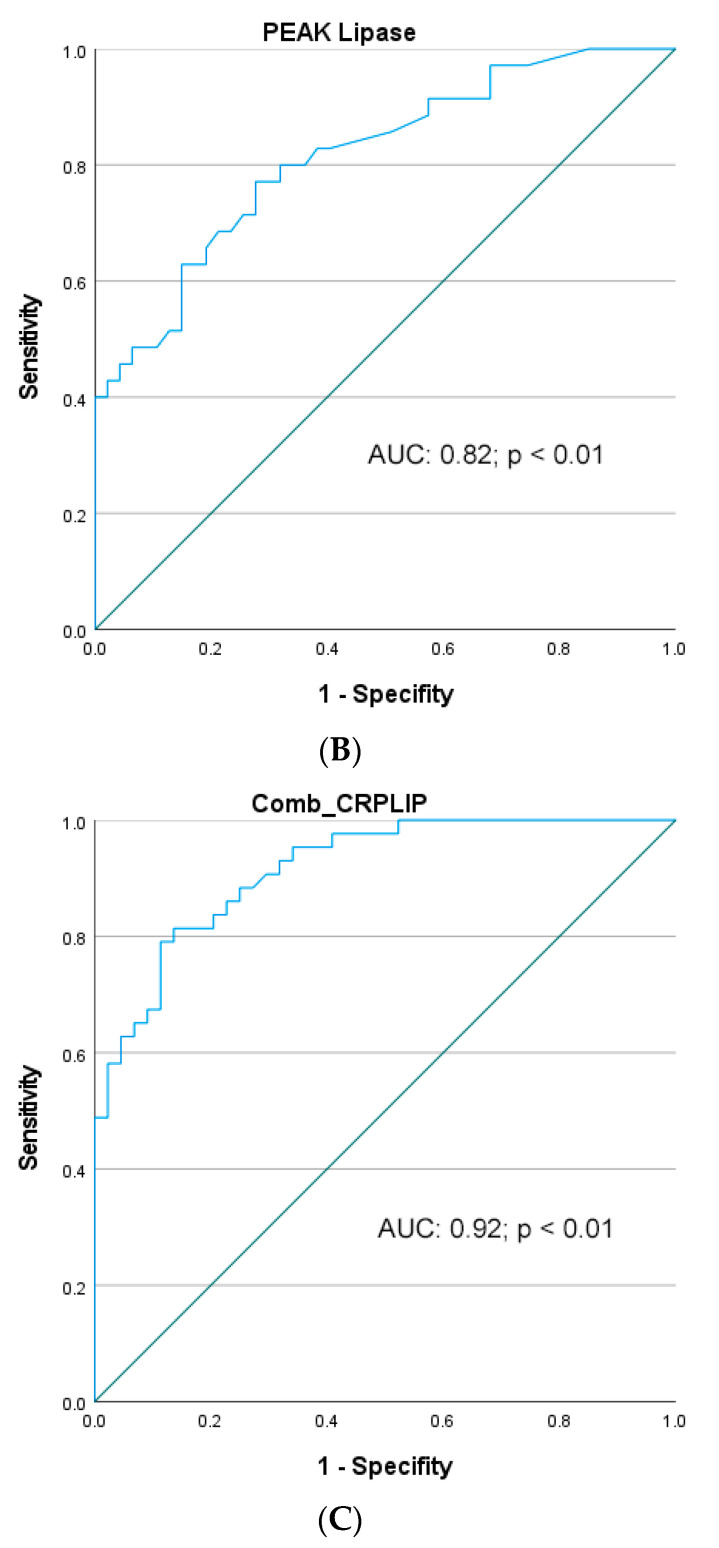
Receiver operating characteristic curve for (**A**) peak CRP value, (**B**) peak lipase value as well as (**C**) combination peak CRP and lipase (Comb_CRPLIP) as predictors for early pancreatic graft dysfunction following SPKT.

Concerning ePGD, the diagnostic accuracy—based on the AUCs obtained from ROC plots—of WBC, PCT, PLR and NLR was fair and moderate (AUC < 0.8), whereas the accuracy of peak CRP and serum lipase levels were excellent (AUC > 0.8). As depicted in Figure 2, the AUC for peak serum lipase was 0.82 (95% CI: 0.72–0.98; *p* < 0.01) with an ideal cut-off of 168 IU/L (sensitivity and specificity: 77% and 78%, respectively), and the AUC for peak CRP was 0.87 (95% CI: 0.81–9.95; *p* < 0.01) with an ideal cut-off 131 ng/mL (sensitivity and specificity: 82 and 87%, respectively) (Figure 2). When peak serum lipase and peak CRP levels were combined (expressed as peak lipase × peak CRP levels/2), the predictive discriminative power was 0.92 (95% CI: 0.85–0.99; *p* < 0.01; sensitivity and specificity: 90 and 89%, respectively) with a valid goodness-of-fit test (HLT: *p* = 0.974; chi square: 3.21) (Figure 2C). On the contrary, with regard to eRGD, the diagnostic predictive accuracy of all analyzed serological biomarkers was fair and moderate (all AUC < 0.8).

### 3.4. Uni- and Multivariate Analysis of Predictive Risk Factors for Early Pancreatic Graft Dysfunction Following SPKT

The relationship between preoperative characteristics, intraoperative factors, postoperative biochemical markers and the development of ePGD following SPKT is shown in Table 6. following significant factors in univariable analysis with deteriorated prognosis and ePGD could be found: age (*p* < 0.01), time on dialysis pre-transplant (*p* = 0.016), no pre-emptive transplantation (*p* = 0.027), donor age (*p* = 0.02), donor BMI (*p* > 0.01), donor cerebrovascular disease as cause of donor death (*p* < 0.01), donor length of ICU stay (*p* = 0.038), cold ischemia time of the pancreas graft (*p* = 0.013), peak CRP levels (*p* < 0.01), peak serum lipase level (*p*< 0.01), serum lipase level on POD 1 > 220 IU/L (*p* < 0.01) and on POD 2 > 150 IU/L (*p* < 0.01) as well as serum CRP level of 180 ng/mL or greater on POD 2 (*p* < 0.01) and >150 ng/mL on POD 3 (*p* < 0.01).

In multivariable analysis (see Table 7), no pre-emptive transplantation (OR 2.4 (95% CI: 1.41–4.01), *p* = 0.021), increased donor age (OR 1.07 (95% CI: 1.03–1.14), *p* < 0.010), donor BMI (OR 1.32 (95% CI: 1.01–1.072), *p* = 0.04), donor cerebrovascular disease as the cause of death (OR 7.8 (95% CI: 2.21–26.9), *p* < 0.01), donor length of ICU stay (OR 1.27 (95% CI: 1.08–1.49), *p* < 0.01) and CIT of the pancreas graft (OR 1.07 (95% CI: 1.03–1.14), *p* < 0.01) could be identified as independent clinical factors associated with increased ePGD following SPKT. Additionally, the following biochemical serum markers including the peak of serum CRP levels (OR 1.12 (95% CI: 1.02–1.23), *p* < 0.01), peak of serum lipase levels (OR 1.04 (95% CI: 1.02–1.07), *p* < 0.01), serum lipase concentration on POD 2 > 150 IU/L (OR 2.9 (95% CI: 1.2–7.2), *p* = 0.021) and serum CRP levels of ≥180 on POD 2 (OR 3.6 (95% CI: 1.54–8.34), *p* < 0.01) and >150 ng/mL on POD 3 (OR 4.5 (95% CI: 1.7–11.4), *p* < 0.01) were revealed as independent predictive factors for ePGD after SPKT. HLT shows a valid goodness-of-fit of the logistic regression model (chi square: 4.572; *p* = 0.561).

## 4. Discussion

Ischemia reperfusion injury (IRI) and subsequent early graft dysfunction play a central role for early as well as long-term outcome and graft function, not only in pancreas transplantation [1,2]. Well-considered monitoring of specific serological biomarkers could be a helpful tool for early detection and diagnosis, treatment choice and induction as well as outcome prediction after transplantation with the final goal of predicting the individual’s risk of allograft injury and ischemic damage, resulting in individualized treatment approaches and outcome improvement after solid organ transplantation [30,31]. Our study provides a comprehensive summary of the predictive values of a panel of widely used global serum biomarkers for the detection of early graft dysfunction for both the pancreas and the kidney graft, respectively, in patients after simultaneous pancreas–kidney transplantation (SPKT). Hereby, peak serum lipase and peak serum CRP as well as their combination showed the most consistent results with good and excellent predictive values (AUCs > 0.8 und > 0.9, respectively), demonstrating a high predictive accuracy in the prediction of ePGD of all analyzed biomarkers in our current study. In contrast, with regard to the prediction of early renal graft dysfunction, the predictive value and diagnostic accuracy of all analyzed serological markers had lower sensitivity and were only assessed as fair to moderate (all AUC < 0.8). According to the findings of our study, the assessment of specific laboratory biomarkers combined with other pre-, intra- and postoperative donor- and recipient-related clinical factors (e.g., donor age and BMI, CIT of the pancreas) may present helpful insights, in order to identify patients at a high risk of ePGD and to predict long-term graft function and outcome, especially of the pancreas graft.

Despite constant improvement in the field of SPKT, the pancreas graft especially remains highly prone to IRI and early graft dysfunction, which leads to early graft damage and consecutively severe patient morbidity and mortality. Due to the high susceptibility of the pancreas graft, unavoidable procedural and surgical steps during the process of transplantation may lead to considerable damage of the graft and impaired outcome [1,31,32].

In this context, the pancreatic allograft is particularly susceptible to injuries in the early reperfusion period with damaging effects on the grafts’ microvasculature resulting in early and severe post-transplant pancreatitis as well as the formation of thromboses in the pancreas vein, in some cases within a few hours after transplantation [14,15,33]. These complications not only significantly impair graft function and survival but are additionally associated with severe surgical complications such as enteric leakage of the anastomosis, profound hemorrhage due to disintegration of vascular anastomosis and profound pancreatitis with fatal outcome for the affected patient [19,34].

Monitoring of serum biomarkers, repeated imaging studies and implementation of clinical scoring systems are widely used for the assessment of mortality and severity of acute pancreatitis in the non-transplant setting [14,16,35,36,37,38]. However, little is known about the usefulness and value of different serum biomarkers for the prediction of specific pancreas graft-related outcomes and early graft dysfunction (mainly ischemia reperfusion injury) after clinical SPKT. In this context, the majority of the implemented scores for non-transplant pancreatitis are impractical for immediate use after transplantation due to various reasons, including but not limited to, a natural increase in used serum biomarkers such as serum lipase and amylase concentrations after transplantation, not necessarily associated with disease severity. Therefore, current research focusses on the detection and verification of new rapid-onset serum biomarkers to predict early pancreatic graft dysfunction as well as ischemia reperfusion injury and following complications after SPKT accurately and promptly, in order to assess the severity of post-transplant pancreatitis as soon as possible, which again may be important for prudent and yet speedy clinical decision making in choice and induction of treatment.

In the past, serum CRP and interleukin levels have been well evaluated for the assessment of the severity and prognosis of acute pancreatitis in “non-transplant” patients [14,16,38]. Recently, a meta-analysis carried out by van den Berg and colleagues showed a superiority of serum IL- 6 within the first 72 h for the early prediction of severity of acute pancreatitis, followed by increased serum CRP levels with adequate predictive values [14]. Another systematic review and meta-analysis could show that serum CRP levels on POD 4 were highly effective (AUC of 0.86, sensitivity and specificity of 86% and 69%, respectively) in predicting pancreas-related complications following pancreas surgery [39]. Intriguingly, our current study of patients after SPKT could reveal that serum CRP levels—especially peak serum CRP levels during the first 3 days after transplantation—are a robust predictive marker with good AUCs > 0.8 for predicting the degree and severity of ePGD. Our findings are consistent with a previous publication, which could demonstrate that peak serum CRP levels correlate well with ePGD and IRI and the consecutive impairment of microcirculation in the early reperfusion period after SPKT, and that an elevation of serum CRP in the early phase after transplantation is associated with increased pancreas graft-associated complications [3]. However, one may argue that the exclusive monitoring of serum CRP levels after SPKT may not be sufficiently organ-specific in monitoring for ePGD and IRI complications due to the rather unspecific nature of serum CRP, which is also increased in the case of any other reason of inflammation, systemical infection and/or renal graft injury after transplantation. Nonetheless, systemical infections, such as pneumonia, blood stream or wound infections, are not very common in the early phase (POD 1–3) after solid organ transplantation and mostly become relevant on POD 4 and later in the case of a prolonged ICU stay. In contrast, ePGD and other pancreatic IRI-associated complications, such as post-transplantation pancreatitis usually develop during the first few days after SPKT, being accountable for early increases in serum CRP levels [34,35]. Therefore, according to the expected time frame of serum CRP rise and in the absence of other clinical signs of early post-transplant infection, elevated CRP levels within the first 72 h after SPKT represent a fairly specific marker for pancreatic tissue injury due to ePGD- and IRI-related complications. Of interest, the results of our ROC analysis concerning CRP course and peak of CRP were less sensitive (all AUC < 0.8) for the prediction of eRGD following SPKT.

Transient elevation of serum pancreatic enzymes in the early postoperative period after SPKT is more common and generally caused by IRI and ePGD, which could reflect the degree of graft-related complications including pancreatitis [36,40,41]. In the non-transplant setting, most previous studies could demonstrate that elevated serum pancreatic enzymes (particularly on POD 1 and 3) as well increased drain fluid pancreatic amylase activity (on POD 1 and 2) are highly predictive parameters for pancreas-related complications following pancreatic surgery [42,43,44,45]. Consistently, the results of our current study show that a serum lipase value of >150 IU/L on POD 2 is an independent prognostic parameter in multivariate logistic regression analysis for ePGD following SPKT. Interestingly, the peak of serum lipase values with a cut-off value >168 IU/L shows excellent predictive correlations (>0.8 AUC) for ePGD and pancreatic graft-related complications after SPKT. On the other hand, the serum lipase values on POD 1 and 2 only had acceptable predictive accuracy (<0.8 AUC) for predicting ePGD.

Based on these former findings and particularly with regard to the results of our ROC analyses, we believe that the combination of monitoring peak serum CRP and serum lipase levels during the first three to five days after SPKT may be very useful for the monitoring of ePGD-associated complications as well as in the prediction of early graft function and long-term outcome in SPKT recipients.

In contrast, the ability of WBC as well as PLR and NLR to predict ePGD and pancreas graft-associated complications as well as early renal graft dysfunction in our patient cohort was assessed as fair. Our current findings are consistent with previous studies in the non-transplant setting, showing weak or acceptable predictive values of PLR and/or NLR for prediction of postoperative pancreas-related complications following pancreatic surgery as well as a reduced ability for the prediction of the severity of the disease in acute pancreatitis [9,14,16,37,43,46]. However, after liver and kidney transplantation alone, preoperative NLR and PLR levels as well as their distinct postoperative course could be identified as independent predictors of early graft dysfunction and decreased graft survival in the long-term follow up [47,48,49,50].

In question of our results concerning serum PCT values, this marker is widely reported as a useful biomarker to differentiate sepsis- and non-sepsis-related inflammatory response and to predict severe bacterial infection as well as to guide discontinuation of antibiotic treatment. The role of PCT and its ability to predict disease severity as well as reliable prognosis in acute pancreatitis in the non-transplant setting, is a matter of dispute [38]. So far, previous studies showed a low predictive accuracy for the severity of disease, prognostic stratification as well as (pancreas related) complications following pancreatic surgery in the setting of acute pancreatitis [9,14,16,37,38,39]. These previous findings are in accordance with our current results in patients after SPKT, only showing a weak correlation and a low predictive accuracy of serum PCT levels in predicting ePGD- and pancreas-related complications after SPKT. Therefore, our data do not support serum PCT as useful in postoperative monitoring of ePGD-associated complications; however, we highly recommend the monitoring of this marker for the early detection of postoperative bacteria-derived infectious complications after SPKT.

Current literature provides only sparce evidence for organ-specific biomarkers for the discrimination between IRI and early graft dysfunction of the pancreas and kidney graft, respectively. Due to the unique characteristic of SPKT, two organs of a very different kind and nature are transplanted simultaneously, of which the pancreas graft is by far the most susceptible for postoperative early graft dysfunction and failure. The kidney graft on the other hand is in comparison much more robust and much less susceptive to ischemia reperfusion injury and postoperative graft failure compared to the delicate pancreas graft. Concerning SPKT and pancreas transplantation alone, only two studies exist which examined this question in detail [51,52]. Glazunova et al., focused on kidney specific serum markers (such as Cystatin C, NGAL, podocin and OPN) after SPKT, which, however, were not found to be specific for IRI, but only for unspecific acute kidney injury [51]. Fernstad et al., could show that pancreatic-specific protein (PASP) is a good marker for early pancreatic graft dysfunction and IRI-associated pancreatitis following SPKT [52]. In a recent review by Prudhomme et al., the findings of preclinical studies with regard to machine perfusion showed promising approaches for the evaluation of pancreas-specific markers in the sense of metabolic assessment of perfusate solutions (Insulin, ATP, LDH, multiple metabolomics) or cell death activity (e.g., caspase, cell-free DNA) during pancreas preservation, which might be implemented for clinical use after successful evaluation in prospective clinical studies in the [1]. Newer approaches, such as the evaluation of non-invasive liquid biopsy and liquid biopsy-based biomarkers, e.g., different protein-panels and extracellular vesicles (EVs), miRNA and cell-free DNA, have shown promising results concerning diagnostic insight in various disorders, not only in malignancies and autoimmune diseases, but also in the evaluation of graft dysfunction after solid organ transplantation as well [53,54,55]. Since the introduction of multi-omics techniques, which are shedding light on discrete genomic, transcriptomic, proteomic and metabolomic signatures, new biomarkers have been the focus of interest in the prediction of allograft outcomes [56]. In this proactive approach, the focus lies on the prediction and prevention of pathological processes by providing earlier and more extensive information than traditional ones [54,57]. In the case of kidney transplantation alone, some metabolic- and protein-associated markers (namely KIM-1, Cystatin C, NAG, NAGL, chemokine (CXCL 9 and 10) or L-FAB) have recently been identified in some clinical studies as potential and “organ-specific” markers for monitoring graft function and detecting early graft dysfunction and IRI-associated complications and outcome after KTA [54]. Furthermore, few current convincing studies identified cell-free DNA (circulating/donor-derived) as well as EVs, specifically kidney-specific parameters (aquaporin, CD133, clusterin, PODXL, SYT17 as well as multiple mRNA signatures), as promising markers for early graft dysfunction in several clinical KTA settings [54,55]. Notably, in the field of genomic and transcriptomic profiling, the assessment of DNA methylation seems to become a potential new and sensitive clinical biomarker in the detection of early graft dysfunction during KTA and consecutively targeting therapy for the future [58]. However, further research is needed to evaluate the significance of these new approaches for early graft dysfunction in SPKT, with the pancreas-specific lipase and alterations of CRP being by far the most specific IRI and graft dysfunction markers for the pancreas graft yet.

Apart from the diagnostical pathway for the detection of early graft dysfunction and IRI-associated complications, for instance by the screening and monitoring of serological biomarkers in combination with clinical risk-factor-dependent prediction scoring systems, therapeutical approaches are the key pathways for reducing IRI-associated damages with encouraging early and long-term success following solid organ transplantation [2]. In this context, conditioning is a broad term generally used to describe strategies to attenuate IRI and early graft dysfunction by inducing biochemical changes within the recipient and transplant allograft. Depending on timing and application, it can be referred to as pre-, peri- and post-conditioning [32,59]. Herein, ischemic preconditioning and remote ischemic preconditioning have previously been shown to induce beneficial effects in solid organ transplantation including SPKT [60]. Insights into the molecular pathophysiology of IRI and subsequent early graft dysfunction have opened the door to new therapeutic targets and novel interventions, such as succinate removal, ferroptosis inhibitors, regulation of complement cascade and manipulation of regulatory cells, such as myeloid-derived suppressor cells (MDSC) and hematopoietic stem cells (HSC), which may play an important role in reducing early graft dysfunction after transplantation [2]. As long-term preservation technologies of the graft, such as machine perfusion, become more and more accessible, the possibility of pharmacological organ conditioning as well as recipient conditioning (e.g., anesthetic conditioning, pharmacological (such as α-Lipoic Acid substitution) and manipulation with novel strategies such as RNA interferences, become more attractive [1,2,8,11,61]. In this regard, differential regulation of early graft dysfunction and IRI-related miRNAs may lead to improved graft function and survival as well as an expansion of the donor organ pool [2].

Although our current study shows promising results, several limitations are important to discuss.

Firstly, the low number of patients in each group and the retrospective, non-randomized design of our current study must be considered, before extrapolating our results to common clinical practice. Therefore, as a future study approach, a detailed multiple logistic regression analysis within a larger cohort should be investigated.

Secondly, although this study represents the results of a big German pancreas transplant center with equivalent surgical procedure and compact and robust follow-up data, the long investigation period as well as different anesthesiologic and operative teams and styles may have had an impact on diagnostical and therapeutical decision.

Thirdly, although in our analysis a variety of the most commonly used global serologic markers of inflammation (CRP, WBC, PCT, NLR, PLR) were evaluated, these—except perhaps for pancreatic-specific lipase—cannot distinguish by laboratory chemistry alone between the source of IRI, hence between pancreas- and kidney-specific injury. Therefore, future large prospective multi-center studies are needed to introduce new and reliable sets of graft-specific serological markers (e.g., miRNAs, cell-free DNA, EVs, etc.), which may identify which organ is affected by IRI and to assess internal/external validity and reliability of the presented data.

## 5. Conclusions

Early and accurate identification of patients with IRI at risk of developing graft-related complications and especially ePGD as well as eRGD after SPKT is needed to guide clinical decision making and to select patients for novel prophylactic treatment. In our current study, we showed that the combination of peak serum CRP and lipase levels are excellent predictive parameters for the monitoring and detection of early pancreatic graft dysfunction and for predicting IRI-associated pancreas-specific clinical outcome following simultaneous pancreas and kidney transplantation. In contrast, the predictive value of these parameters for early renal graft dysfunction was less specific. The resulting risk groups according to proposed marker cut-off levels allow for improved stratification and better outcome compared to established clinical and paraclinical criteria such as CIT or special donor variables. Therapeutic approaches including different ways of conditioning the graft or recipient for the amelioration of graft-specific IRI aimed to improve prognosis and outcomes in solid organ transplantation. The multifactorial molecular pathophysiology in the setting of IRI in transplantation medicine requires a multimodal approach. Therefore, the most preferable approach is the integration of both pharmacological and technical preconditioning techniques applied to the donor, the donor organ and the organ recipient. Hereby, pharmacological conditioning such as the regulation of miRNAs or complement cascade in combination with machine perfusion seems a promising approach. These novel therapeutical possibilities in combination with strong diagnostical biomarkers and prognostic clinical variables for the prediction of early graft dysfunction may be used in future studies, evaluating new ways to improve impaired microcirculation following pancreas transplantation.

## Figures and Tables

**Table 1:** Baseline perioperative transplant characteristics of recipients and donors according to the status of early pancreatic graft dysfunction (ePGD).

Variables	All (*n* = 105 Patients)	ePGD- (*n* = 62 Patients)	ePGD+ (*n* = 43 Patients)	*p*-Value
**Donor**				
Age, years	24.32 +/− 11.6	22.9 +/− 11.2	28.1 +/− 12.9	0.020
Gender, male/female	62 (59)/43 (41)	37 (60)/25 (40)	25 (58)/18 (42)	0.874
BMI, kg/m^2^	22.6 +/− 4.2	21.5 +/− 3.5	24.1 +/− 3.2	<0.01
Katecholamine use	75 (71)	43 (69)	32 (74)	0.377
ICU-LOS, days	3.4 +/− 3.8	2.8 +/− 2.5	4.5 +/− 4.2	0.030
Hypertension, *n* (%)	11 (10)	7 (11)	4 (9)	0.792
Cerebrovascular disease	33 (31)	25 (40)	8 (19)	0.018
Cardiac reanimation	9 (8.6)	3 (4.8)	6 (14.3)	0.060
**Recipient**				
Age, years	42.9 +/− 9.1	40.9 +/− 8.7	46.1 +/− 8.6	<0.01
Gender, male/female	58 (55)/47 (45)	36 (58)/26 (42)	22 (51)/21 (49)	0.484
BMI, kg/m^2^	25.1 +/− 4.2	25.3 +/− 4.2	24.5 +/− 4.3	0.291
HbA1c, (%)	7.8 +/− 1.7	7.6 +/− 2.5	8.1 +/− 1.8	0.125
Duration of Diabetes, years	26.9 +/− 8.5	27.2 +/− 8.3	25.1 +/− 8.9	0.090
Comorbidities				
Cardiovascular disease, *n* (%)	31 (30)	16 (25)	15 (36)	0.256
Peripheral Arterial Disease, *n* (%)	17 (16)	7 (11)	10 (24)	0.084
Hypertension, *n* (%)	84 (80)	50 (79)	34 (81)	0.842
Number of antihypertensive medications	2.3 +/− 1.9	2.7 +/− 1.5	2.2 +/− 1.6	0.210
Previous dialysis, *n* (%)	80 (76)	45 (73%)	35 (81)	0.296
Duration of dialysis, months	31.9 +/− 32.6	35.1 +/− 29.9	26.8 +/− 35.6	0.273
Waiting time, months	8.3 +/− 11.4	9.3 +/− 12.4	6.9 +/− 9.5	0.300
**Transplant characteristics**				
CMV D+/R-	20 (19)	13 (21)	7 (16)	0.621
HLA Mismatches > 2/6	75 (71)	45 (73)	30 (70)	0.752
Immunosuppression				
Induction therapy (ATG/SRL/None)	69 (66)/25 (24)/11 (10)	39 (62)/17 (27) 7 (11)	30 (71)/8 (19)/4 (10)	0.584
CNI, FK506/CsA	96 (91)/9 (9)	59 (94)/4 (6)	37 (88)/5 (12)	0.319
AP drug, MMF/SRL/none	87 (83)/15 (14)/3 (3)	53 (84)/9 (14)/1 (2)	34 (81)/6 (14)/2 (5)	0.631

**Table 2:** Intra- and postoperative outcome and function associated with early pancreatic graft dysfunction (ePGD) following simultaneous pancreas–kidney transplantation.

Variables	All (*n* = 105 Patients)	ePGD- (*n* = 62 Patients)	ePGD+ (*n* = 43 Patients)	*p*-Value
Cold ischemia time, hours				
Pancreas	11.1 +/− 2.6	10.5 +/− 2.6	11.9 +/− 2.4	0.010
Kidney	12.1 +/− 3.1	11.4 +/− 2.6	12.2 +/− 3.3	0.194
Warm ischemia time, minutes				
Pancreas	38.1 +/− 9.1	37.2 +/− 9.4	39.5 +/− 8.5	0.225
Kidney	36.9 +/− 10.9	37.7 +/− 11.2	35.8 +/− 10.4	0.454
Operating time, minutes	383 +/− 34	378 +/− 89	386 +/− 118	0.808
Delayed graft function (%)				
Kidney	18 (17)	7 (11)	11 (26)	0.049
Hospital stay	42.5 +/− 20.6	29.5 +/− 24.8	62.3 +/− 34.9	0.030
CMV Infection	21 (20)	14 (23)	7 (16)	0.427

**Table 3:** Pre-and postoperative WBC, CRP, lipase, NLR and PCT levels stratified by early pancreatic graft dysfunction (ePGD).

Time after SPKT															
	Preoperative			POD 1			POD 2			POD 3			POD 5		
Variables	ePGD-	ePGD+	*p*-Value	ePGD-	ePGD+	*p*-Value	ePGD-	ePGD+	*p*-Value	ePGD-	ePGD+	*p*-Value	ePGD-	ePGD+	*p*-Value
WBC (×109/L)	8.1 +/− 3.4	7.2 +/− 2.2	0.152	14.3 +/− 3.3	18.2 +/− 5.2	0.01	12.5 +/− 3.4	15.2 +/− 5.2	0.03	11.5 +/− 4.1	14.5 +/− 6.3	0.05	10.8 +/− 4.2	13.2 +/− 5.6	0.113
CRP (mg/L)	5.8 +/− 4.4	9.6 +/− 12.1	0.305	85 +/− 63	110 +/− 56	0.04	121 +/− 45	219 +/− 89	<0.01	102 +/− 34	238 +/− 26	<0.01	56 +/− 23	105 +/− 59	0.04
Lipase (IU/L)	62 + 12	58 +/− 23	0.671	250 +/− 356	565 +/− 226	<0.01	123 +/− 84	360 +/− 130	0.02	51 +/− 79	176 +/− 99	0.04	57 +/− 61	101 +/− 182	0.231
NLR	2.9 +/− 2.1	2.8 +/− 3.8	0.789	9.4 +/− 2.3	15.10 +/− 5.6	0.01	8.2 +/− 3.1	18.2 +/− 4.9	<0.01	5.9 +/− 2.7	13.2 +/− 4.8	<0.01	3.8 +/− 5.2	8.3 +/− 2.6	0.08
PLR	123.4 +/− 15.8	135.2 +/− 28.2	0.456	212.4 +/− 32.9	269.9 +/− 45.3	0.03	178.6 +/− 56.7	199.8 +/− 88.2	0.567	151.9 +/− 56.3	172.2 +/− 69.4	0.856	144.8 +/− 49.2	161.4 +/− 23.8	0.789
PCT (ng/mL)	3.2 +/− 5.6	2.8 +/− 3.2	0.876	77.3 +/− 50.8	98.1 +/− 86.5	0.496	69.4 +/− 34.3	143.9 +/− 56.3	0.02	39.4 +/− 37.8	99.6 +/− 16.7	0.131	15.1 +/− 16.3	38.5 +/− 56.3	0.06

**Table 4:** ROC analysis of different serum markers for the prediction of early pancreatic graft dysfunction following simultaneous pancreas–kidney transplantation.

Days	Variables	Cut-Off Values	AUC (95% C)	*p*-Value	Sensitivity	Specifity
Preoperative	CRP	3.9	0.51 (0.37–0.63)	0.712	58	51
	Lipase	54	0.53 (0.41–0.68)	0.546	51	63
	PCT	3.1	0.5 (0.58–0.94)	0.946	35	81
	WBC	6.75	0.43 (031–0.54)	0.258	63	42
	NLR	3.12	0.69 (0.58–0.78)	0.215	80	78
	PLR	121	0.64 (0.60–0.73)	0.07	55	34
POD 1	CRP	102	0.62 (0.49–0.74)	0.06	61	70
	Lipase	211	0.71 (0.58–0.82)	0.03	70	71
	PCT	44.1	0.71 (0.51–0.96)	0.06	90	63
	WBC	12.4	0.46(0.25–0.58)	0.08	74	53
	NLR	10.4	0.67 (0.62–0.7.8)	0.624	87	56
	PLR	215	0.69 (0.59–0.78)	0.129	81	72
POD 2	CRP	179	0.80 (0.74–0.93)	<0.01	84	71
	Lipase	159	0.79 (0.68–0.9)	0.019	78	64
	PCT	45.8	0.69 (0.45–0.887)	0.115	72	82
	WBC	13.9	0.61 (0.48–0.72)	0.09	58	42
	NLR	13.8	0.73 (0.69–0.82)	0.184	53	88
	PLR	175	0.57 (0.51–0.64)	0.173	58	61
POD 3	CRP	135	0.80 (0.69–0.88)	<0.01	81	79
	Lipase	112	0.65 (0.52–0.77)	0.123	82	79
	PCT	38.7	0.79 (0.61–0.96)	<0.01	78	79
	WBC	14.3	0.62 (0.51–0.74)	0.453	56	72
	NLR	9.6	0.65 (0.61–0.72)	0.821	57	84
	PLR	148	0.70 (0.62–0.79)	0.245	72	51
POD 5	CRP	61	0.74 (0.63–0.86)	<0.01	56	88
	Lipase	66	0.49 (0.35–0.64)	0.456	42	67
	PCT	27.3	0.80 (0.59–0.99)	0.083	71	90
	WBC	9.75	0.52 (0.42–0.61)	0.152	61	38
	NLR	5.9	0.57 (0.51–0.62)	0.09	57	95
	PLR	139	0.66 (0.57–0.73)	0.345	75	68
Peak CRP (ng/mL)	PCRP	131	0.87 (0.81–0.95)	<0.01	82	87
Peak Lipase (lU/L)	PLIP	168	0.82 (0.72–0.98)	<0.01	77	78
Combination Peak Lipase × Peak CRP/2	Comb_CRPPLIP	145	0.92 (0.85–0.99)	<0.01	90	89

**Table 5:** ROC analysis of different serum markers for the prediction of early renal graft dysfunction following simultaneous pancreas–kidney transplantation.

Days	Variables	Cut-Off Values	AUC (95% C)	*p*-Value	Sensitivity	Specifity
Preoperative	CRP	4.5	0.42 (0.27–0.55)	0.228	52	49
	Lipase	29	0.43 (0.26–0.59)	0.222	85	25
	PCT	1.5	0.58 (0.49–0.82)	0.456	42	75
	WBC	5.600	0.49 (0.33–0.59)	0.444	84	29
	NLR	2.7	0.63 (0.53–0.74)	0.016	79	51
	PLR	79	0.66 (0.56–0.77)	0.09	77	52
POD 1	CRP	135	0.58 (0.49–0.65)	0.08	76	51
	Lipase	168	0.70 (0.57–0.83)	<0.01	76	64
	PCT	55.9	0.61 (0.32–0.89)	0.484	67	48
	WBC	16.8	0.56(0.42–0.69)	0.370	37	90
	NLR	13.3	0.73 (0.69–0.82)	0.05	57	76
	PLR	167	0.80 (0.71–0.89)	0.04	74	84
POD 2	CRP	145	0.79 (0.68–0.87)	0.03	71	76
	Lipase	126	0.63(0.48–78)	0.09	49	79
	PCT	63.8	0.64 (0.38–0.89)	0.234	57	55
	WBC	8.75	0.48 (0.36–0.62)	0.835	89	19
	NLR	11.4	0.77 (0.73–0.88)	0.07	66	82
	PLR	135	0.67 (0.56–0.78)	0.231	49	72
POD 3	CRP	112	0.71 (0.64–0.79)	0.123	80	56
	Lipase	60	0.55 (0.38–0.71)	0.557	49	69
	PCT	31.4	0.72 (0.68–0.91)	0.09	67	79
	WBC	11.7	0.59 (0.52–0.83)	0.567	68	47
	NLR	5.2	0.61 (0.58–0.69)	0.234	47	63
	PLR	114	0.67 (0.59–0.79)	0.567	72	60
POD 5	CRP	45	0.65 (0.58–0.80)	0.234	64	54
	Lipase	52	0.44 (0.29–0.60)	0.410	43	69
	PCT	20.9	0.79 (0.72–0.99)	0.06	78	82
	WBC	11.9	0.43 (0.30–0.59)	0.123	39	59
	NLR	2.5	0.51 (0.49–0.68)	0.110	72	82
	PLR	99	0.57 (0.44–0.71)	0.245	52	71
Peak CRP (ng/mL)	PCRP	118	0.8 (0.71–0.87)	<0.01	82	65
Peak Lipase (lU/L)	PLIP	165	0.75 (0.71–0.88)	0.06	78	69

**Table 6:** Univariate logistic regression analysis of factors correlated to the occurrence of early pancreatic graft dysfunction following SPKT.

Variables	Odds Ratio (95% CI)	*p*-Value
Recipient age, years	1.07 (1.02–1.12)	<0.01
Recipient gender (female versus male)	1.21 (0.55–2.65)	0.631
Recipient BMI, per 5 kg/m^2^	0.94 (0.85–1.05)	0.289
Time on dialysis pre-transplant, months	0.97 (0.95–0.99)	0.016
Pre-emptive transplantation (no versus yes)	2.71 (1.37–5.39)	0.027
Duration of diabetes mellitus, years	0.95 (0.91–1.07)	0.09
Recipient peripheral arterial disease (yes versus no)	2.5 (0.86–7.29)	0.09
Recipient cardiovascular disease (yes versus no)	1.63 (0.69–3.81)	0.258
HLA mismatches >2 versus <2	2.1 (0.9–3.3)	0.08
Induction therapy (no versus yes)	1.18 (0.32–4.33)	0.759
Donor stay length in the intensive care unit, days	1.19 (1.01–1.33)	0.038
Donor cardiac arrest (yes versus no)	3.3 (0.8–14.1)	0.09
Donor age, years	1.04 (1.0–1.01)	0.025
Donor gender (female versus male)	0.97 (0.43–2.1)	0.935
Donor BMI, per 5 kg/m^2^	1.3 (1.1–1.6)	<0.01
Donor cerebrovascular disease as cause of death (yes versus no)	6.97 (3.81–12.7)	<0.01
Cold ischemia time pancreas, h	1.23 (1.04–1.45)	0.013
Cold ischemia time kidney, h	1.09 (0.95–1.25)	0.196
Warm ischemia time pancreas, min	0.97 (0.92–1.02)	0.210
Warm ischemia time kidney, min	0.98 (0.94–1.02)	0.450
Peak CRP levels	1.14 (1.03–1.26)	<0.01
Peak lipase levels	1.02 (1.01–1.03)	<0.01
Lipase POD 1; >220 IU/L	3.7 (1.61–8.8)	0.002
Lipase POD 2; >150 IU/L	2.91 (1.30–6.54)	0.009
CRP POD 2; >180 mg/L	3.6 (1.54–8.34)	<0.01
CRP POD 3; >150 mg/L	4.5 (1.7–11.4)	<0.01
PCT POD 1; >45 ng/mL	1.38 (0.31–6.13)	0.098
PCT POD 3; >25 ng/mL	2.1 (1.1–7.6)	0.060

**Table 7:** Multivariate logistic regression analysis of factors correlated to the occurrence of early pancreatic graft dysfunction following SPKT.

Variables	Odds Ratio (95% CI)	*p*-Value
Recipient age, years	1.04 (0.98–1.14)	0.167
Time on dialysis pre-transplant, months	1.01 (0.98–1.04)	0.279
Pre-emptive transplantation (no versus yes)	2.4 (1.41–4.01)	0.021
Donor age, years	1.07 (1.03–1.14)	<0.01
Donor BMI, per 5 kg/m^2^	1.32 (1.01–1.72)	0.04
Donor cerebrovascular cause of death (yes versus no)	7.8 (2.21–26.9)	<0.01
Donor stay length in the intensive care unit, days	1.27 (1.08–1.49)	<0.01
Cold ischemia time pancreas, hours	1.15 (1.02–1.55)	0.021
Warm ischemia time pancreas, minutes	0.95 (0.89–1.02)	0.110
Peak CRP levels	1.12 (1.02–1.23)	<0.01
Peak lipase levels	1.04 (1.02–1.07)	0.013
Lipase POD 1; >220 IU/L	2.3 (0.87–6.19)	0.090
Lipase POD 2; >150 IU/L	2.9 (1.21–7.13)	0.021
CRP POD 2; >180 mg/L	3.6 (1.54–8.34)	<0.01
CRP POD 3; >150 mg/L	4.5 (1.7–11.4)	<0.01

## Data Availability

Our database contains highly sensitive data that may reveal clinical and personnel information about our patients and lead to their identification. Therefore, according to organizational restrictions and regulations, these data cannot be made publicly available. However, the datasets used and/or analyzed in the current study are available from the corresponding author upon reasonable request.

## Abbreviations

IRI	Ischemia reperfusion injury
ePGD	Early pancreas graft dysfunction
eRGD	Early renal graft dysfunction
SPKT	Simultaneous pancreas–kidney transplantation
CRP	C-reactive protein
WBC	White blood cell count
PCT	Procalcitonin
NLR	Neutrophil–lymphocyte ratio
POD	Postoperative days
ROC	Receiver operating characteristics
AUC	Area under the curve
KTA	Kidney transplant alone
ICU-LOS	Intensive care unit length of stay
BMI	Body mass index
PAD	Peripheral arterial disease
HLA	Human leukocyte antigen
CMV	Cytomegalovirus
DGF	Delayed graft function
ATG	Antithymoctye globulin
CNI	Calcineurin inhibitor
MMF	Mycophenolate mofetil
SRL	Sirolimus
IL2-RA	Interleukin receptor antagonist
ANOVA	Analysis of variance
CI	Confidence interval
OR	Odds ratio
HLT	Hosmer–Lemeshow test
EVs	Extracellular vesicles
PASP	Pancreatic-specific protein
MDSC	Myeloid-derived suppressor cells
HSC	Hematopoetic stem cells
